# Collectanea

**Published:** 1828-10

**Authors:** 


### COLLECTANEA.

Floriferis ut apes in saltibus omnia libant,
Omnia nos, itidem, depascimur aurea dicta.

### PHYSIOLOGY.

***Transposition of the Viscera.***—In the Annales de la Med. Phys. (May 1828,) there is an account of a curious transposition of some of the viscera of the chest and abdomen. The subject of this phenomenon was a soldier, who had always enjoyed good health, but was killed in a duel. On inspecting the body, the liver was found in the left hypochondrium, and the spleen in the right. The liver was unusually pale; the gall-bladder very much distended with bile; the spleen was smaller and denser than usual. The cardiac opening of the stomach was turned to the right side, the pyloric to the left. In the chest, the heart was found occupying the right side of the thoracic cavity; and the peculiarities usually seen in the left lung here existed in the right. The man seemed to have been aware of something peculiar about him, for he often joked with his comrades, saying he was sure his heart was on the right side, whatever the faculty might say.

***Case of Rumination.***—In the same Journal (for April,) there is a case of rumination detailed. A young man, æt. seventeen, an armourer by trade, of strong constitution and sanguine temperament, from the age of nine, he had always felt the food he had taken, rise up into his mouth, about half an hour after swallowing it. He used to chew it over again, and swallow it the second time, without either pleasure or disgust. If ever he tried, by an effort of the will, to prevent this singular rising up of his food, he was sure to bring on pain in the epigastric region. This lad is always hungry, and eats indifferently animal or vegetable food: he eats quickly, and sometimes, after eating, has the sensation of slight bitterness in the mouth. He has frequent colics of short duration, and is troubled with procidentia recti. If he drinks spirituous liquors, they are subjected to the same process of rumination. He enjoys perfect health.

---

*Inspiration of Inflammable Gas.*—This experiment was made by Signor GIACOMO CARDONE, in consequence of the difference of opinion on the effects of this gas on the lungs, entertained by SCHEELE, FONTANA, and others. The air being expelled from the lungs as much as possible, the mouth-piece of a bladder, containing thirty cubic inches of the gas, was applied to the mouth, and the gas inhaled at two inspirations. An oppressive difficulty of respiration, and a distressing constriction at the pit of the stomach, were the first sensations; these were followed by abundant perspiration, a general tremor over the whole body, seeming to commence at the knees; an extraordinary sense of heat, slight nausea, and violent headache. "My eyes," says Sig. G., "beheld things but indistinctly, and a deep murmuring sound was in my ears. After a short time, all these effects ceased, except that of heat, which increased in an alarming manner; but ultimately, by the abundant use of cold drinks, I was restored to my original state of health."—*Giornale di Fisica.*

---

### PATHOLOGY.

*Case of Sanguineous Discharge from the Breasts.* By Dr. JACOBSON.—A woman, twenty-four years old, had enjoyed a very good state of health, excepting that she was frequently subject to bleedings from the nose, and determinations of blood to the head and chest. She married at the age of fourteen. She menstruated the following year for the first time, and continued perfectly regular. At the menstrual period, she always suffered considerable pain in the belly. At the age of seventeen she became pregnant, and during the first two months the menses continued regular. They then ceased; but reappeared on the sixth and seventh month, with the usual suffering. She went through her labour without any untoward symptoms.

Two months after her confinement, although she suckled her infant, the menstrual discharge returned. At this period her mind was much disturbed, and she was attacked with a very severe illness, at the commencement of which she had, during three or four days, a discharge of blood from the nails of both hands, and from the gums. In the course of some time she perfectly recovered.

She suckled for two years, the secretion of milk remaining plentiful, and the menses regular. When she weaned the infant, the secretion of milk still continued very abundant. It flowed almost constantly and freely from the breasts, and, when it ceased for a short period, she complained of pain and tension in the chest. Her health did not suffer from this continued discharge of milk. The breasts remained soft, and she still menstruated regularly.

She now suckled the child of a neighbour for eighteen months; and, whenever she had an opportunity, she gave the breast to other children, to relieve herself as much as possible from her inconvenient burthen.

She continued in this state for six years, when a physician promised to cure her. She was successively bled, at short intervals, from both arms, the forehead, and from behind the ears. Immediately afterwards the secretion of milk ceased, and in its place a discharge of blood took place from both breasts, with violent pains extending towards the shoulders and neck. Night and day, with the exception of a very short cessation, this discharge of dark-coloured blood continued. It tinged the linen of a very deep colour, and gave it a fetid smell. It remained the same in quantity and quality during the periods of menstruation. In other respects the woman was in good health. The digestive powers were not deranged when she was free from pain. The breasts were so tender to the touch, that the slightest pressure upon them was insupportable. During either rainy or cold weather, when the discharge from the breasts diminished, the patient suffered severely from pains, nausea, and vomiting. She was soon attacked with hematemesis, and discharge of blood from the lungs. These symptoms were, however, relieved by acids and cooling drinks.

Leeches were now frequently applied to the vulva, and blood was taken from the feet; digitalis, prussic acid, and aperients, together with pediluvia, were also employed; the breasts were supported by a suspensory bandage, and they were carefully protected from every irritation. The disease, however, resisted every remedy.

After each discharge of blood from the lungs and stomach, she quickly recovered; but she remained subject to violent pains and spasms of the stomach, and vomiting from the slightest irregularity of diet or any mental exertion.—*Journal Complementaire.*

---

*Observations on the Anatomy and Diseases of the Kidneys and Ureters.* By J. Bouillaud, d.m.p.—Neither the structure nor pathological conditions of the kidneys have been so much attended to as other internal parts. M. Andral, to whose pathological labours we are so much indebted, has passed over this part of the subject in silence. Even Morgagni has given but an imperfect sketch of it. In some subjects but one kidney is found. M. B. has met with one instance of this kind. The kidney was situated across the spine, and was furnished with two ureters: it was considerably larger than the ordinary size.

*Lobulated kidneys.*—M. B. has seen this conformation in four adult bodies. The external configuration of the kidneys resembled in some degree the hemispheres of the cerebrum. The lobes and sinuosities which refracted them represented the circumvolutions and infractions of the brain. In one instance, two ureters proceeded from the right kidney, and, at the termination of about two inches, they united into one canal. The left kidney was naturally formed.

*Hypertrophy of the kidneys.*—M. B. has frequently met with this affection, and generally in only one kidney. It is recognised by the following appearances: The kidney is a quarter, or a third, or perhaps even one-half, larger than the natural size. Its substance is firmer, more compact, and redder. It is probable that in such cases the renal artery is enlarged, although this fact has not been determined. Hypertrophy of the kidney occurs under the influence of various causes, which determine to it an unusual quantity of blood. The most likely circumstance to produce this kind of plethora in one kidney is the existence of some obstruction to the passage of the blood towards the other. It happens, consequently, that hypertrophy of one kidney is frequently detected when the other is in a state of atrophy. Hypertrophy of the heart, and of the external muscles, takes place equally under the same conditions which preside over the increased size of the kidney.

*Atrophy, or diminished nutrition of the kidneys.*—This disease M. B. has frequently seen. Its characters are diametrically opposite to those of hypertrophy of the organ. The size of the kidneys is less than natural; their substance is paler; they contain less blood, and appear shrunk. Whatever cause obstructs the current of blood to the kidneys may produce an atrophy of them. In every such instance, M. B. has been able to demonstrate a greater or less obstruction to the free circulation of the blood. The pressure of an enlarged spleen has sometimes produced atrophy of the left kidney. The right kidney has been similarly affected by the continued, yet gradual, pressure of an enlarged liver. In other organs, as the heart, the lungs, the breast, the testicle, pressure frequently causes the same diminution of size.

*Infiltration of urine, and cysts of the kidneys.*—M. B. observes that no pathologist has hitherto described this affection; it is not, however, very rare, but it may easily escape the observation of a careless practitioner; the following are the characters of it: on the surface of the kidneys may be seen several round vesicles, which raise the covering membrane of these organs. These vesicles appear to be small cysts in the substance of the kidney, and are probably formed by a certain quantity of urine, which has distended the uriniferous tubes, in consequence of some obstruction to the passage of the fluid. M. B. has seen some of these cysts as large as a cherry. Sometimes, instead of numerous vesicles, he has detached one large sac, which he presumed to have been formed from the union of several smaller ones, of which the parietes had ruptured. He has found the whole of the kidney transformed into one large sac, containing either a transparent serous, or turbid, fluid.

*Inflammation of the kidneys, and of the disorganizations which follow inflammation.*—In consequence of their peculiar structure, the kidneys do not easily become the seat of those disorganizations which result from inflammation. Nephritis is marked by the following appearances: redness, tumefaction, presence of pus, softening of the structure of the organ, abcesses, ulceration of the external surface, conversion of the parenchymatous substance into a tuberculous, or encephaloid matter, which is, in a great measure, the product of the diseased secretion of the affected kidney. Cysts, either on the surface, or in the substance of the kidney, may result from inflammation. In two or three cases, M. B. has found the kidney converted into a fatty yellowish substance. The symptoms of the various ulcerations which the kidneys occasionally undergo are very obscure; this circumstance will not be considered so extraordinary when we consider, first, that the deep seated situation of the kidney embarrasses our examinations; secondly, that derangement of the function of the kidneys produces similar symptoms to those which result from various affections of the bladder and ureters; thirdly, that pain is by no means a constant attendant upon renal disease. If we are to rely upon the statement of most pathologists, acute pain is the almost inseparable attendant upon inflammation of the kidneys; it is not denied that such is frequently the case, but M. B. affirms that he has observed the most decided marks of renal inflammation in the bodies of patients, who had never complained of pain in the region of the kidneys. This absence of pain may be more easily conceived, when we reflect that the kidneys in a natural state are but slightly sensible.

Violent pain is not seldom complained of in the region of the kidneys, when no disease of them is to be detected. The presence of a certain quantity of blood or pus in the urine, when there exists no disease of the bladder, is a symptom of some affection of the kidney; when to this symptom is united a smart attack of fever, the existence of nephritis may be strongly presumed. At the commencement of the disease, if both kidneys are affected, an almost total suppression of urine takes place. Chronic nephritis, like most other internal inflammations of a chronic character, produces a slow fever, which destroys the patient by throwing him into that state termed renal consumption. When the affected kidney continues the performance of its functions, the urine is much altered in its appcarance, but sometimes it ceases to secrete; the urine being formed only by the healthy kidney, presents no unusual appearance, and the diagnosis of the disease is then extremely difficult. If both kidnies are simultaneously disorganised, so that a total cessation of the secretion of urine takes place, the same phenomena will occur as we observe in animals, in which both ureters are tied, or both kidneys removed, violent fevers quickly arises, and a strong smell of urine is exhaled from the body. Is hypertrophy of the kidneys ever the cause of diabetes? M. B. is not furnished with sufficient facts to justify him in giving a positive answer to this question, but he has observed hypertrophy of the kidneys where the patient had been affected with diabetes. The ureters, like all other parts of the body may suffer from inflammation, and undergo various alterations of structure in consequence; their canals may be much enlarged, diminished, or entirely obliterated; dilatation of the ureter may arise from any cause which obstructs the free passage of the urine into the bladder; contraction or obliteration may follow from any accidental compression from inflammation of the internal membrane which lines the cavity, or from the cessation of the passage of the urine through the canal, from the function of the kidney being no longer performed in consequence of disease. The symptoms of affections of the ureters are as obscure as those which attend diseases of the kidneys. If both canals are obliterated at the same time, death would speedily result; but if one ureter only is obstructed, the calibre of the other will be increased considerably, from the additional duty which it will have to perform under such circumstances. In support of these observations, M. Bouillaud details several interesting cases.—*Ibid.*

### SURGERY.

*Case of urinary calculus lodged in the interior of the glans penis, and removed by incision.*—Dr. Schwartz, in the Journal of Græfe and Walther, mentions a case of this kind: A young naturalist, of lively temperament, in 1807, felt, in making water, that a rough and unequal body traversed the canal of the urethra, and fixed itself at the base of the glans; he was then nineteen years of age. As he was little incommoded by this, and nothing could be felt to the touch, he had nothing done for it. In the years 1814, 18, 19, he passed, at times, gravel with his water, and sometimes with great pain. The patient, not very attentive to his health, disregarded the increased size and hardness of the glans penis, believing it to be natural. In 1822, he married, and could perform all the duties of a husband without pain; he had children. Three years after marriage, the ardor urinæ, which he had for a long time felt, increased much, and sometimes drops of blood followed. Violent neuralgic symptoms made him consult a medical man in 1826, but no careful examination took place. Other symptoms, such as loss of appetite and sleep, depression of spirits, a wasting of flesh, and inflammation in the prepuce, induced him to consult a surgeon. Having subdued the inflammation of the prepuce, he was sounded, and a hard body was felt at the end of the urethra, situated in the fossa navicularis. The accompanying history left no doubt of the nature of the mischief: a directory was introduced before the calculus, the upper part of the glans, as well as the prepuce, were divided by a cut of the bistoury, and the lips of the wound being separated, a calculus, occupying the whole of the glans, was seen, and was easily extracted; the whole of the cavity was slightly ulcerated. The operation was accompanied with less pain than making water latterly had been. It healed up perfectly; and, in fifteen days, he was able to resume his avocations, and since has enjoyed excellent health. Although the whole tissue of the glans was destroyed, and after the operation there remained only two thin folds of skin, yet it assumed a kind of natural appearance; but, in actu coitus, he thought the voluptuous feeling was diminished. The calculus had the form of a chesnut, solid, and was of a dirty white colour, with some red points. The circumference of the base, which was nearly circular was three inches seven lines; the greatest diameter was one inch and half a line; from the base to the top was one inch. Some days after the operation, the calculus weighed 284 grains.

---

### CHEMISTRY.

*Citric Acid from Gooseberries.*—M. Tilloy, by the annexed process, has prepared citric acid from gooseberries, so as to be able to obtain it for twelve francs, ninety-six centimes the kilogramme; whereas the price of citric acid in France is from twenty-nine to thirty francs for the same weight.

The gooseberries are to be bruised and fermented; the alcohol is to be separated by distillation; the residuum is to be pressed, to extract the whole of the liquid. To this liquor, while hot, carbonate of lime is to be added as long as effervescence takes place: after standing, the citrate of lime is to be collected, and suffered to drain; it is to be repeatedly washed, and then pressed. The citrate of lime thus obtained, being still coloured and mixed with malate of lime, is to be mixed with water to the consistence of a thin syrup, and is then, while hot, decomposed with sulphuric acid, diluted with twice its weight of water. The liquid resulting from this operation is a mixture of sulphuric (malic?) and citric acid, and is to be again treated with carbonate of lime. The precipitate, when collected on a filter, is to be plentifully washed, pressed, and again mixed with sulphuric acid; the clear liquor, containing the acid, is to be decolorized by animal charcoal, and evaporated. When it is sufficiently concentrated, it is suffered to deposit, and the clear liquor poured off is put into stoves heated from 20° to 25° Centig. Coloured crystals are thus obtained, which are to be drained, slightly washed, and recrystallised.—*Journal de Pharmacie.*

---

*Blue Colour by the action of Muriatic Acid upon Albumen.*—Various unsuccessful experiments appear to have been made to produce this blue colour; first observed, we believe, by M. Caventou. According to M. Robiquet, the more acid employed, the more readily is the blue colour produced, to a certain extent. He finds that seven or eight parts of acid to one part of albumen yield the most intense blue, even at a low temperature; but its development is favored by a temperature of 25° to 30° Centig.—*Ibid.*

---

*Decomposition of Ammonia by Metals.*—M. Savart found that 141.91 grains of thin copper wire became 142.382 grains, or acquired an increase of 0.472 in weight, when used for four hours to decompose ammonia. As the wire was in a slight degree oxidised, the experiment was repeated; and, when every precaution was employed, the increase amounted to $\frac{1}{275}$; and 0.105 of an unknown substance was absorbed by the copper, and its specific gravity was diminished from 8.8659 to 7.7919.

Iron also increases in weight, and diminishes in specific gravity, by similar treatment, and will strike fire with flint like ordinary steel.—*Ann. de Chim.*

---

*Corydalin, a new Vegetable Alkali.*—According to M. Wackenroder, this alkali is contained in the root of the fumitory, (not the common fumitory, Fumaria Officinalis, but the Fumaria Cava, and Corydalis Tuberosa of Decandolle.) The dry root is to be coarsely powdered, and digested for some days in water; filter the infusion, and precipitate with excess of potash; dry the precipitate, and treat it with boiling alcohol until it ceases to dissolve any thing. It sometimes happens that, during the cooling of the alcohol, crystals of corydalin are deposited. The solution is to be evaporated to dryness, and the residuum is to be dissolved in weak sulphuric acid: this solution is then to be decomposed by an alkali, either caustic or carbonated. A white deposit is formed, which by drying becomes of a light gray colour.

Dry corydalin soils the fingers very much; it is insipid and inodorous. It is soluble in alcohol; and this solution, when hot and saturated, deposits colourless prismatic crystals of a line in length. By slow spontaneous evaporation, fine laminæ are formed. The solution acts as an alkali upon vegetable blue colours. At a temperature below that of boiling water, it melts into a deep green-coloured fluid, which, when solidified, has a crystalline texture, and is transparent in thin laminæ. At a higher temperature, it yields water and ammonia, and is converted into a transparent brown mass. Ether dissolves corydalin with the same facility as alcohol; caustic potash dissolves it in considerable quantity.

This alkali forms extremely bitter salts with acids; sulphuric acid forms two different salts; one which crystallises is obtained when the acid is digested with excess of base; the solution is to be filtered and evaporated: the product is very slightly soluble in water. When a small quantity of sulphuric acid is added to a solution of corydalin in alcohol, so as not to saturate the base perfectly, a portion of crystalline matter is deposited, and there remains a stratum of a greenish transparent substance, which is unalterable by exposure to the air, and readily soluble in water; the solution reddens litmus paper slightly; an excess of acid renders it purple, and eventually blackens it. Nitric acid, when diluted and cold, dissolves and forms a colourless solution with corydalin; but, when heated, it becomes of a red colour, which, when the solution is concentrated, becomes of a blood-red. This action is so strong that, by the aid of heat, the smallest quantity of corydalin may be discovered in a fluid. Muriatic acid forms with this alkali an uncrystallisable salt; acetic acid is still more difficult of combination with it than sulphuric acid; but it forms a crystalline salt, which may be redissolved a second time in water, and crystallised. Tannin is one of the most sensible tests of corydalin, as for all other vegetable bases. The precipitate is white when the solution is dilute, and grayish yellow if concentrated.—HENSMAN's *Repertoire de Chimie.*

### MISCELLANEOUS.

*Proportion of Male and Female Children.*—M. BAILLY, of the French Institute, has lately made a series of observations connected with the subject of the relative births of male and female children. From exact registers kept in one locality, it appears, he says, that there were more female conceptions than male conceptions in the months of March and July; and these two months, he observes, are, the first on account of the reoccurrence of warmth, and the second on account of the heat of the weather, the part of the year least favorable to the activity of the generative powers, at least with a view to fecundation.

*On the Species or Varieties in the Human Race.*—LINNÆUS, in his Systema Naturæ, divided men into four varieties, according to the colour of the skin; giving each variety the name of the part of the world where it was most common. DUMÉVILLE considers that there were six distinct varieties, which he names—1, Caucasian, or European Arabs; 2, Hyperborean; 3, Mongolian; 4, American; 5, Malay; 6, Ethiopian. CUVIER reduced the number of varieties to three. VIZEY, in his History of Man, divided the genus into two species, according to the facial angle, noting three varieties and sub-varieties to each species. DESMOULINS has lately further divided the genus man into eleven species; and BORY SAINT VINCENT, in a very elaborate paper on the varieties and species of this genus, has added four other species to this extended list; and has given the peculiarities, habits, manners, and appearances of each of the species, and an account of their probable origin. He divided the genus into two sections: the first he called Leiotrichi, or smooth-haired men, which he again subdivided into those which are peculiar to the old world, as, 1, Homo Japeticus, the sons of Noah, which he divided into several races; 2, Homo Arabicus, the Arabs; 3, Homo Indicus, the Hindoos; 4, Homo Scythicus, the Scythians; 5, Homo Sinicus, the Chinese. Secondly, those smooth-haired men which are common to the old and new world, as, 6, Homo Hyperboreus, the Laplanders; 7, Homo Neptunianus, the Malays and New Zealanders; 8, Homo Australasius, the New Hollanders. Thirdly, the straight-haired men which are peculiar to the new world, as, 9, Homo Columbicus, the Carribees; 10, Homo Americanus, the Americans; and, 11, Homo Patagonicus, the Patagonians. The second section he designates by the name of Ouloitrichi, or crisped-haired men, usually called negroes. The white varieties of this tribe are not known. 12, Homo Œthiopicus, the Ethiopians; 13, Homo Cafer, the Cafre; 14, Homo Melaninus, the Cochin Chinese; and, 15, Homo Hottentottus, the Hottentots.—*Ann. Phil.*

*White Cats with Blue Eyes, deaf.*—It is stated, in the Magazine of Natural History, that white cats with blue eyes are always deaf. A gentleman had a white Persian cat: she produced various litters, and of her offspring some were entirely white, and these were invariably deaf; but others were mottled, and all those which had the least speck of colour had the faculty of hearing as usual.

